# Early Path Nursing on Neurological Function Recovery of Cerebral Infarction

**DOI:** 10.1515/tnsci-2019-0028

**Published:** 2019-10-02

**Authors:** Ling Chen, Zhena Han, Junjie Gu

**Affiliations:** 1 Outpatient emergency treatment, Xixi branch of Zhejiang university hospital, Zhejiang, China; 2 Department of rehabilitation, Hangzhou first people's hospital, Zhejiang, China; 3 Emergency Department, Hangzhou first people's hospital, Zhejiang, China

**Keywords:** early path rehabilitation nursing, cerebral infarction, neurological function

## Abstract

**Purpose:**

to study the application of path type early rehabilitation nursing in the nursing of patients with cerebral infarction and to explore its impact on the recovery of neurological function.

**Methods:**

Patients with acute cerebral infarction in our hospital were randomly divided into two groups. The control group used conventional treatment methods. The experimental group used path type early rehabilitation care based on conventional treatment methods and observed the curative effect.

**Results:**

The NIHSS scores in the experimental group were significantly lower than those in the control group, and the P value was less than 0.05, which was statistically significant.

**Conclusion:**

Path type early rehabilitation nursing has a positive effect on the treatment of patients with cerebral infarction, which contributes to the recovery of neurological function of patients and is worthy of promotion in treatment.

## 1 Introduction

Cerebral infarction is caused by sudden decrease or stop of blood flow in the local blood supply artery, resulting in ischemia, hypoxia, tissue necrosis and softening of the brain tissue in the blood supply area. Then there are clinical signs and symptoms of neurological deficits such as hemiplegia and aphasia in the corresponding sites. Cerebral infarction seriously endangers people’s lives and health, and brings enormous and heavy mental, material and economic burdens to patients, families and society [1]. With the continuous development and advancement of medical science in China in recent years, the etiology and pathology of cerebral infarction have been studied in depth. Therapeutic techniques such as drugs and interventions and the level of rescue for critically ill patients have been greatly improved, and the mortality rate has been significantly reduced [2]. However, most of the surviving cerebral infarction patients have dysfunctions such as exercise, sensation, speech and cognition to varying degrees. It causes a great psychological burden on the patient and leads to a serious decline or even loss of labor capacity, which seriously affects the quality of life and quality of survival. With the aging of China’s population and lifestyle changes, age, smoking, obesity, alcohol, diabetes, high blood skin, commercial lipemia, hyperhomocysteinemia and other risk factors for cerebrovascular accidents are increasing. This has greatly increased the incidence of cerebral infarction [3]. Clinical pathway refers to a standardized work, which is a specific process. Its main movement is in the measurement of common diseases and multiple diseases. In the process of nursing, the staff of our department mainly formulate, adjust and execute nursing programs for patients through responsible nurses and rehabilitation teachers according to the specific conditions of patients. Planned and individualized nursing program can improve patient compliance and rehabilitation effect, which can also make nurses more reliable in the process of work, so as to avoid blind nursing of nurses.How to prevent and treat various neurological dysfunction after cerebral infarction is an important issue faced by the majority of rehabilitation workers. In this context, the effects of path-based early rehabilitation nursing on the recovery of neurological function in patients with cerebral infarction are studied in this paper.

It firstly describes the current research status of cerebral infarction and its neurological rehabilitation nursing, and then selects patients with acute cerebral infarction in our hospital and randomly divides into two groups. The control group used conventional treatment methods. The experimental group used path type early rehabilitation care based on conventional treatment methods and observed the curative effect. Finally conclusions are drawn.

## 2 Related work

Based on the theoretical basis of the plasticity of the brain, the purpose of rehabilitation training is to stimulate the damaged body through external movements and sensations, so that it can restore the lost function to adapt to the functional changes of new life and work. In recent years, rehabilitation training on the neurological function of cerebral infarction has become the focus of many scholars.

Araki Y *et al* showed that rehabilitation training can promote transient changes in neurological activity during exercise, strengthen the activity of the cerebral cortex, increase the thickness of the cerebral cortex and protein content, and promote the formation of new blood vessels [4]. Nagai K *et al* pointed out that early rehabilitation can also regulate the expression of inflammatory factors and reduce the nervous system inflammation, which can more effectively promote the recovery of neurological function in patients with acute cerebral infarction. Rehabilitation can promote the connection of axons in the brain of the patient, and realize the establishment of collateral circulation and recombination of the contralateral brain, and effectively exert the residual function of the brain, which makes the motor function and self-care ability of the patient recover quickly [5]. Studies by Gao J *et al* have also shown that there is a time-related rehabilitation-mediated cortical reorganization after cerebral infarction. Compared with the late rehabilitation group, the early rehabilitation group can better preserve the topographic map of the cortex. Rehabilitation training in the early stages of brain death can stimulate the function of the remaining cortex (the ipsilateral and contralateral side of the infarcted tissue). Moreover, if the intervening time of rehabilitation is prolonged, the sensitivity to this stimulus will decrease [6]. Boji BM *et al*. pointed out that within two weeks after stroke, peripheral and contralateral areas of the infarcted lesion will temporarily produce some cytokines and proteins related to nerve repair. Many factors that promote nerve growth will change within a few weeks. The expression of these factors not only enhances the remodeling function of the brain, but also prompts the brain to respond most to rehabilitation during this period [7]. Lin Z *et al*. observed the effects of early rehabilitation training on brain damage and recovery in mice after cerebral infarction in 77 animal models of middle cerebral artery occlusion. The results showed that compared with the control group, the motor function of the rehabilitation group was significantly improved, and the volume of cerebral infarction was reduced. Moreover, the number of NGF immunohistochemical cells around the lesion was significantly increased, indicating that early rehabilitation training can help the recovery of brain function damage (Lin Z *et al*. 2017) [8]. Animal studies such as Kawahara T showed that the exercise function of the rats with cerebral infarction was improved after early rehabilitation and the infarct size was reduced [9]. Functional magnetic resonance imaging, such as Bae HW, confirms that rehabilitation can promote brain function reorganization [10]. Wan QI *et al* analyzed the changes of cortical functional areas of active and passive movements before and after rehabilitation in patients with partial pain by fMRI. It was found that the complementary functional area of the frontal cortex has a localized excitatory area. The display of the finger movement function area of the affected limb is consistent with its clinical improvement in some patients after receiving systemic rehabilitation [11]. Zhu F *et al* applied fMRI to find that patients with stroke had extensive activation in the ipsilateral and contralateral cerebral cortex activation areas after hand-exercise treatment after mandatory exercise therapy (CIMT). The activation phenomenon was significantly reduced after 2 weeks. The conclusion that use dependence affects cerebral cortical function reorganization is obtained [12]. Yu C and other experiments found that in the structure of the cerebral cortex, the most capable plasticity is the nerve cells in the cortex. Upper limb movement involves numerous nerve cells in the motor cortex [13].

China has made active explorations in continuous rehabilitation care. However, rehabilitation care started late, and there are still some problems due to the relatively tight medical resources. In particular, there are few research results on the effect of path type early rehabilitation nursing on the recovery of neurological function in the nursing of patients with cerebral infarction. Further research is still needed.

## 3 Information and methods

### 3.1 Normal information

112 patients with cerebral infarction who were admitted to our hospital from January 2016 to January 2018 were selected. All patients were admitted to the Department of Neurology, and the patients were randomly divided into two groups, 56 in each group. In the control group, male and female patients were 30 and 26, respectively, with an average age of (62.32 ± 8.77) years. In the experimental group, male and female patients were 28 and 28, respectively, with an average age of (61.98±8.64) years. The data of the two groups were comparable. The inclusion criteria for patients were as follows: diagnosed as ischemic stroke by head CT or MRI; age greater than 18; motor function disorder; GCS score greater than 12. Patients with the following symptoms were excluded: brain stem infarction; cognitive function has been severely impaired; serious complications; onset time is greater than 3d.

### 3.2 Method

Both groups of patients underwent routine care on basic care. After the patient was discharged from the hospital, the responsible nurse made a telephone interview and understood the patient’s condition. The patient was reviewed at 12 weeks and evaluated. On the basis of this, the experimental group also carried out path type early rehabilitation nursing intervention. The specific method of intervention is as follows: Intervention time: The patient is evaluated by the ward’s responsible nursing and medical personnel within 24 hours after admission. It mainly includes physical and neurological conditions, as well as mobility. At the same time, the medical staff, together with the patient and the family, set the goal of treatment and rehabilitation, and proposed specific rehabilitation and treatment methods, and then began to intervene according to the planned plan. It is also necessary to prevent complications during this period. Rehabilitation exercise: Rehabilitation exercise is also carried out according to the path table. On the first day, the body function is evaluated in detail, and the patient is assisted in performing limb movements and joint activities in the bed. At the same time, these actions are taught to patients. Each action is done 5 times, 2 times a day, and the range of motion needs to be less than 90 degrees. During the course of a specific activity, the patient’s head needs to be biased toward the affected side to sense the affected limb. On the second day, the main task was to teach the patient to turn over, followed by Bobath training and bridge movement. Tell the patient during the training process that you should pay attention to your physical experience and pay attention to your studies. Each teaching takes 10 to 20 minutes. The bed is also gradually raised, initially raised by 30 degrees, and then adjusted every 20 degrees, 20 to 30 minutes, up to 90 degrees. Train twice a day. On the third day, the sitting balance exercise was carried out, and the training of the patient’s self-care ability was emphasized. Patients are encouraged to perform autonomous activities according to the specific circumstances of the patient, and such contact is performed twice a day, and each action needs to be repeated 5 times. On the fourth day, the patient’s condition needs to be evaluated. It mainly includes the patient’s muscle strength, muscle tone, and limb flexibility. Then the appropriate treatment of the path of treatment, and then sit and stand training. In this training, when you sit, you need to let the torso lean forward, the center of gravity needs to move to the forefoot, and the weight is done through the patient’s legs. This movement is performed twice a day, and each movement is performed 5 times. The fifth day to the seventh day are mainly training for standing. Let the patient’s feet apart, as wide as the shoulders, and then swing the body to the left and right, mainly to move the center of gravity to the affected side. In the standing position, the patient can hold the hand to the bed for knee flexion. Perform 2 times a day for 10 to 20 minutes each time. Pay attention to the protection of the knee joint of the patient during this process. If the patient can stand for 5 minutes and is relatively stable, the patient can also be encouraged to walk slowly. In the process, if the patient feels flustered and anxious, they should stop in time. On the eighth day, the patient’s physical activity, muscle strength, and muscle tone were observed. Appropriate adjustments were made to the treatment method, followed by lower limb resistance training. During the period from the ninth day to the discharge, pay attention to the evaluation of the patient’s motor function and provide necessary guidance. Responsible nurses need to evaluate the patient daily and use personalized rehabilitation measures. The rehabilitation therapist conducts a consultation on the patient’s condition every week and provides guidance.

### 3.3 Evaluation method

The evaluation was mainly carried out at several time nodes, which were before admission, the second week after the intervention, and the twelfth week. The scale used for the evaluation is the American Stroke Scale (NIHSS). The scale includes eleven items, namely the level of consciousness, upper and lower limb movements, visual field, gaze, etc. The higher the score, the more severe the patient's symptoms.

### 3.4 Data processing

The data processing in this paper is SPSS18.0, which is represented by (x±s) in the measurement process, and the count is expressed by “example (%)”. Perform a P test on the data. A P value of less than 0.05 indicates a significant and statistically significant result. Through rigorous data processing, this study has a certain degree of reliability and validity.

## 4 Result and Discussion

Table 1 is the NIHSS scores for the two groups of patients before and after the intervention. As can be seen from Table 1, the patients in the experimental group scored significantly lower after the intervention than the control group. The P value was less than 0.05 and was statistically significant. In the experimental group, the NIHSS score was lower than that of the control group after 2 and 12 weeks of intervention (P<0.05) (Table 1).

**Table 1:** Two sets of NIHSS scores(score, *x̄* ± *s* )

group	Number of cases	Before intervention	2 weeks	12 weeks
Experience group	56	6.94±3.22	3.33±3.59	1.20±1.55
control group	56	6.95±3.37	5.41±3.36	2.29±1.99
t	—	0.07	2.22	2.39
P	—	0.88	0.03	0.01

The above data show that with the increase of treatment time, the neurological function of patients has been restored. However, in the experimental group, the neurological function of patients has been restored more obviously and the effect is better. This can indicate that early rehabilitation nursing by path can play a positive role in the recovery of neurological function of patients with cerebral infarction. Early rehabilitation intervention can increase the average cerebral blood flow speed, establish collateral circulation, restructure brain function, and alleviate inflammation of nervous system, which may improve the neurological function of patients after stroke.

The study in this paper shows that as the treatment time increases, the patient’s nerve function is restored. However, the neurological function of the experimental group recovered more obviously and the effect was better. This may indicate that path type early rehabilitation care can play a positive role in the recovery of neurological function in patients with cerebral infarction. The clinical pathway refers to a standardized work, a specific process whose main exercise is in the measurement of common diseases and multiple diseases. In the process of nursing, the staff of our department mainly develop, adjust and implement the nursing plan for the patients according to the specific conditions of the patients through the responsible nurses and the rehabilitation teachers. A planned, personalized care program can improve patient compliance and rehabilitation, which can also enable caregivers to be more informed during the work process, so as to avoid blind care of the caregiver. Recovery of brain function in patients is the focus of central nervous system recovery. For the cerebral cortex, the reorganization of function is limited. Functional training helps the development of the cerebral cortex and contributes to the recovery of the central nervous system. Gan Zhaoru and other studies have found that early rehabilitation interventions increase the average blood flow velocity of the brain, and establish collateral circulation, and recombine brain function, and reduce the inflammatory response of the nervous system, which may improve the neurological function of patients after stroke. In summary, path type early rehabilitation care for patients with cerebral infarction can enable caregivers to perform quality care services and help patients recover their nerve function and daily activities as soon as possible.

## 5 Conclusion

Cerebral infarction is a common localized brain dysfunction disease. The blood circulation disorder in the brain causes hypoxia and ischemia in the brain tissue, which eventually leads to localized brain tissue necrosis or brain softening. Based on this, the effects of path-based early rehabilitation nursing on the recovery of neurological function in patients with cerebral infarction are studied in this paper. 112 patients with acute cerebral infarction in our hospital were randomly divided into two groups. The control group used conventional treatment methods. The experimental group used path type early rehabilitation care based on conventional treatment methods and observed the curative effect. The results show that through the training of Bobath, the active activities of patients, and the activities of family members to promote patient activities, the frequency and intensity of rehabilitation training of limbs are effectively increased, and the recovery of limb movement patterns is promoted. After the above rehabilitation nursing measures, the total effective rate of limb function recovery in the observation group was 96.67%, which was significantly higher than that in the control group (83.33%, P<0.05). It is suggested that the nursing group of the observation group can promote the recovery of limb motor function, and the effect is better than the control group. The activity index of daily living and the score of neurological deficit in the observation group were significantly better than those in the control group (P<0.05). It is suggested that the observation group nursing program can significantly improve the patient’s nerve function and improve the ability of daily living activities. This proves that the use of path-based early rehabilitation care for patients with cerebral infarction can significantly promote the recovery of limb motor function, and can improve the neurological function of patients and improve the ability of daily living activities, which is worthy of clinical application. This study was a clinical subject, and the subjects were randomly divided into groups, but failed to achieve double-blind enrollment. More confounding factors have some interference with the study, such as the size of the infarct, the age of the patient, blood pressure, sugar, vascular stenosis and other factors may affect the results of this research topic, which still needs further improvement.

## References

[1] Jneid H (2016). Cardiac Rehabilitation After Myocardial Infarction: Unmet Needs and Future Directions.[J]. Jama Cardiology.

[2] Jang S H, Kwon H G (2016). Recovery of an injured corticospinal tract during the early stage of rehabilitation following pontine infarction[J]. Neural Regeneration Research.

[3] Gao J, Zhang H J (2017). Comparison of effects on dysphagia and psychological state after cerebral infarction between chin tuck against resistance exercise and Shaker exercise[J]. Eur J Phys Rehabil Med.

[4] Araki Y, Furuichi M, Nokura H (2018). [Influence of Pre-existing Cognitive Impairment on Rehabilitation Outcomes in Patients with Cerebral Infarction].[J]. Brain and nerve = Shinkei kenkyū no shinpo.

[5] Nagai K, Yamaguchi F (2017). Improved functional independence measure facilitates return to home afterparalyzed upper-limb training: a case report[J]. Journal of Physical Therapy Science.

[6] Gao J, Zhang H J (2017). Effects of chin tuck against resistance exercise versus Shaker exercise on dysphagia and psychological state after cerebral infarction.[J]. European Journal of Physical & Rehabilitation Medicine.

[7] Boji B M, Ho J (2016). Poster 98 Cerebral Infarction Secondary to Cement Embolism Through a Patent Foramen Ovale After Percutaneous Kyphoplasty: A Case Report[J]. Pm & R the Journal of Injury Function & Rehabilitation.

[8] Lin Z, Wenyu W U, Mingxia W U (2017). Row acupuncture at the gaps between phalanges for dorsal stretch of fingers after cerebral infarction[J]. Chinese Acupuncture & Moxibustion.

[9] Kawahara T, Hagiwara M, Takahashi H (2017). Cerebral Infarction by Paradoxical Gas Embolism During Laparoscopic Liver Resection with Injury of the Hepatic Vessels in a Patient without a Right-to-Left Systemic Shunt:[J]. American Journal of Case Reports.

[10] Bae H W, Kim H D, Choi SW (2016). Acute Cerebral Infarction as a Rare Thrombotic Event in Myelodysplastic Syndrome: A Case Report[J]. Annals of Rehabilitation Medicine.

[11] Wan Q I, Shi M, Liu B (2016). Resting-state Functional Magnetic Resonance Imaging in Application of the Motor Function Rehabilitation in Patients with Acute Cerebral Infarction[J]. Rehabilitation Medicine.

[12] Zhu F, Gao J, Gao R (2017). Clinical efficacy of electroacupuncture combined with motor imagery therapy on hemiplegic cerebral infarction[J]. Chinese Acupuncture & Moxibustion.

[13] Yu C, Wang W, Zhang Y (2017). The Effects of Modified Constraint-Induced Movement Therapy in Acute Subcortical Cerebral Infarction[J]. Frontiers in Human Neuroscience.

[14] Mizutani K, Sonoda S, Wakita H (2016). Effects of exercise and bryostatin-1 on serotonin dynamics after cerebral infarction[J]. Neuroreport.

[15] Tsukasa S, Keisuke H, Hajime N (2016). Clinical Characteristics and Lesions Responsible for Swallowing Hesitation After Acute Cerebral Infarction:[J]. Dysphagia.

